# Kernel density estimation and transition maps of Moldavian Neolithic and Eneolithic settlement

**DOI:** 10.1016/j.dib.2018.01.051

**Published:** 2018-02-03

**Authors:** Robin Brigand, Olivier Weller

**Affiliations:** UMR 8215 (Trajectoires), CNRS & Univ. Paris-I Panthéon Sorbonne, France

## Abstract

The data presented in this article are related to the research article entitled “Neo-Eneolithic settlement pattern and salt exploitation in Romanian Moldavia” (Brigand and Weller, 2018) [1]. Kernel density estimation (KDE) is used in order to move beyond the discrete distribution of sites and to enable us to work on a continuous surface that reflects the intensity of the occupation in the space. Maps of density per period – Neolithic I (Cris), Neolithic II (LBK), Eneolithic I (Precucuteni), Eneolithic II (Cucuteni A), Eneolithic III-IV (Cucuteni A-B and B) – are used to create maps of density difference (Figs. 1–4) in order to analyse the dynamic (either non-existent, negative or positive) between two chronological sequences.

**Specifications Table**

**Table:** 

Subject area	*Archaeology*
More specific subject area	*Dynamic of Neolithic and Eneolithic settlement pattern related to salt springs*
Type of data	*figures*
How data was acquired	*GIS-based processing*
Data format	*Images*
Experimental factors	*N/A*
Experimental features	*N/A*
Data source location	*Moldavia, Romania*
Data accessibility	*The images are available with this article*

**Value of the data**

• Methodology to move beyond the discrete distribution of sites and to enable us to work on a continuous surface that reflects the intensity of the occupation of the space.
• Sites for which the chronological calibration is insufficient are taken into account in the density calculations per period.
• Maps of density difference that allow us to perceive the evolution- non-existent, negative or positive- between two chronological sequences.

## 1 Data

By focusing on the eight districts of Romanian Moldavia, a total area of 46 000 km² delimited on the west by the Eastern Carpathians and on the east by the River Prut, the present study highlights, at a scale, the evolution of territorial choices of the Neolithic and Eneolithic of North East Romania (6000–3500 BCE) regarding salt springs [1]. The salt resources of this region (sodium chloride springs, rock salt outcrops, saline soils), among the most abundant in Europe, have been systematically surveyed and have been the subject of bibliographical and cartographical research by our team since 2005. In this article we concentrate on salt springs of geological origin, directly linked to the deposits in the foothills, and whose use in recent times (for food preservation, fodder and therapeutic purposes) is well attested in ethnographic studies [2].

## 2 Experimental design, materials and methods

Kernel density estimation (KDE) is used in order to move beyond the discrete distribution of sites and to enable us to work on a continuous surface that reflects the intensity of the occupation of the space. The KDE method provides an estimation of the site density, defined by a moving window. The density value obtained takes into account the size of the neighbourhood. This method is well known [3], [4], [5], [6] and has been used for archaeological applications, in particular for intra-site analysis [7].

The choice of the radius (*h*) is an important parameter because *h* determines the degree of smoothing. Using too small a radius will produce an irregular surface that is problematic, especially when the total number of points is relatively small. On the contrary, too large a radius will result in a loss of precision, favouring general trends. In our case, the search radius (bandwidth) is 5000 m.

A major problem emerges when we apply this method to archaeological data: sites for which the chronological calibration is insufficient (24% of the total) are not taken into account in the density calculations per period. Thus, in order to take account of these poorly calibrated sites, we have decided to attribute a weighting in line with the duration of each period. For example, a value of 1 is attributed to a site that is well calibrated. However, poorly calibrated sites in the Cucuteni are attributed a value of 0.45 for the Cucuteni A and 0.55 for the Cucuteni A-B and B. The same protocol is used for poorly calibrated sites in the Neolithic and Eneolithic.

**Fig. 1:**
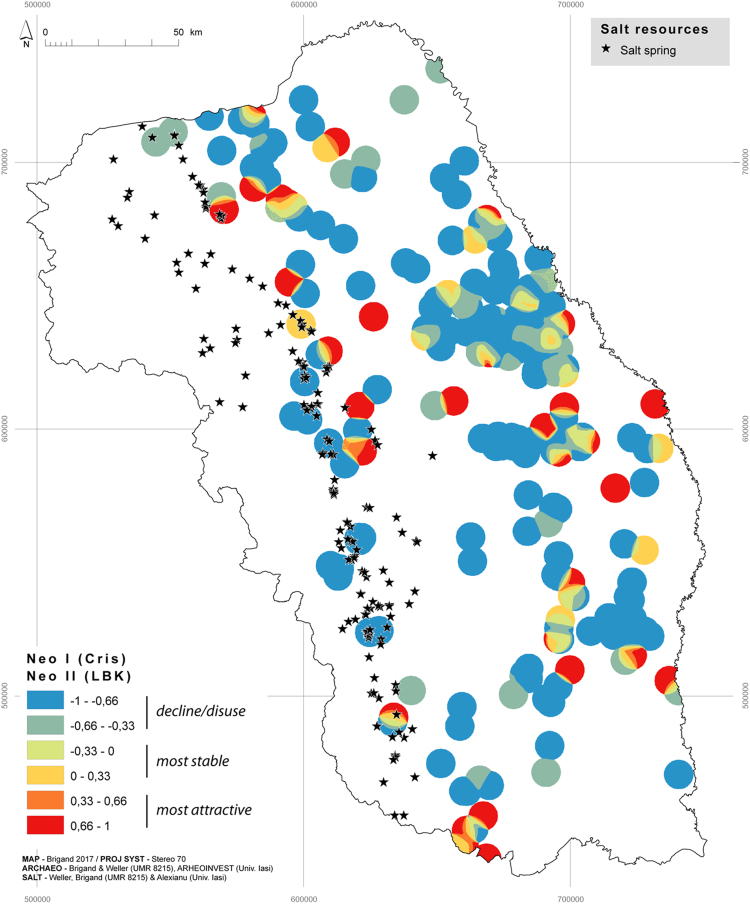
Positive or negative dynamics between Criş, c. 6000–5300 BCE, and Linearbandkeramik (LBK), c. 5300–5000 BCE.

**Fig. 2:**
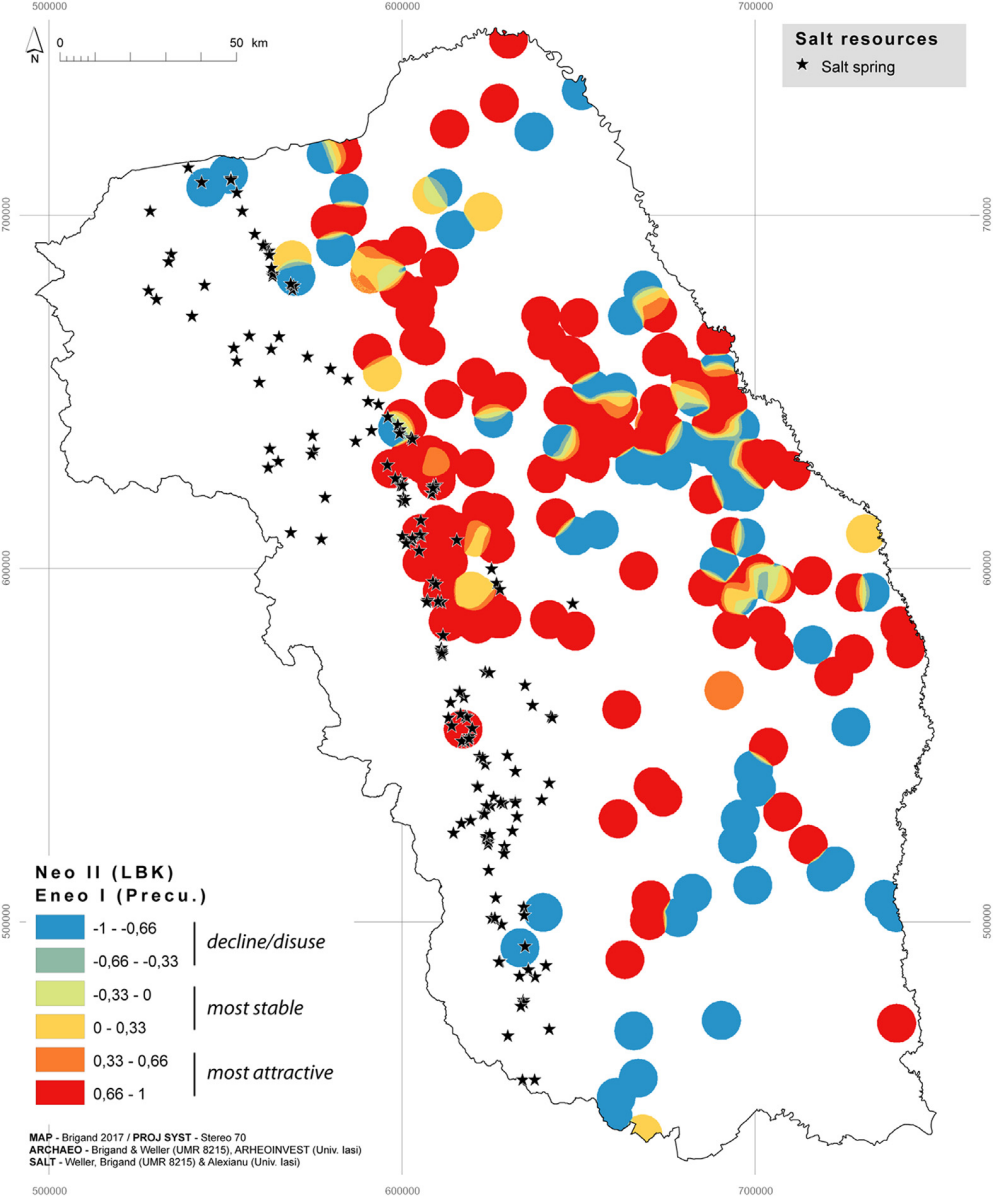
Positive or negative dynamics between LBK and Precucuteni, c. 5000–4600 BCE.

**Fig. 3:**
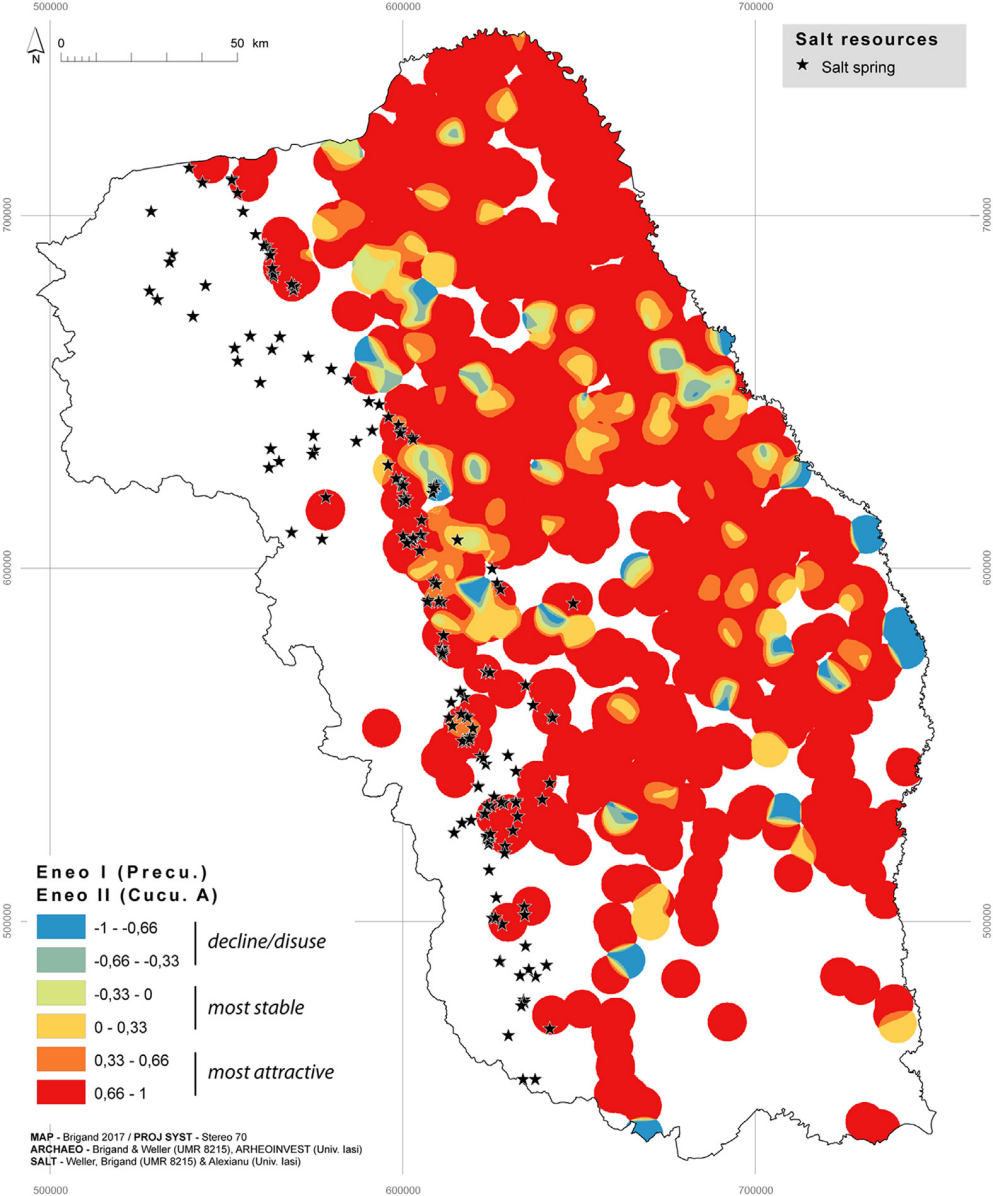
Positive or negative dynamics between Precucuteni and Cucuteni A, c. 4600–4100 BCE.

**Fig. 4:**
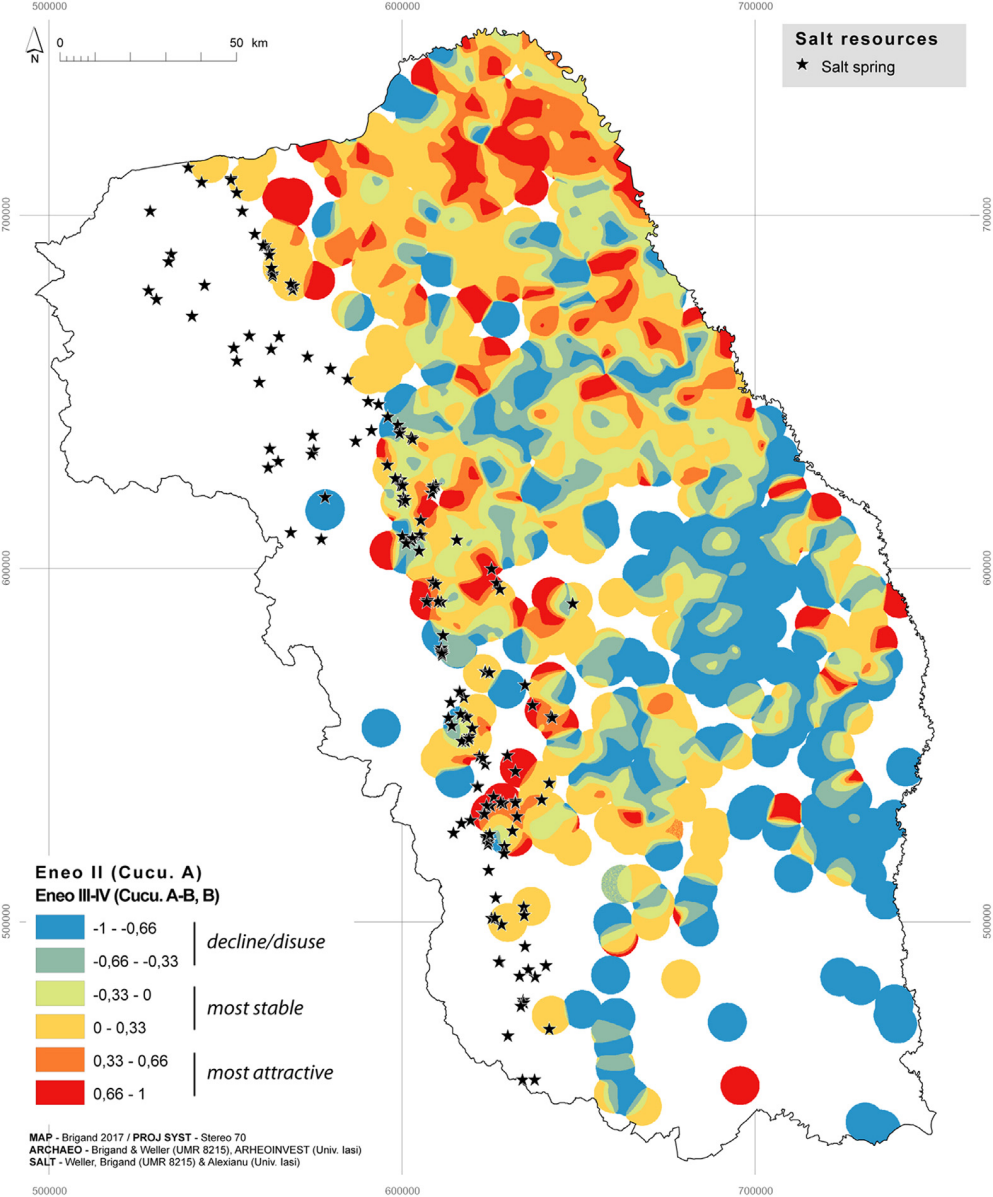
Positive or negative dynamics between Cucuteni A and Cucuteni A-B and B, c. 4100–3500 BCE.

These maps of density per period are used to create maps of density difference that allow us to perceive the evolution- non-existent, negative or positive- between two chronological sequences(Fig. 1, Fig. 2, Fig. 3, Fig. 4). The method is based on map algebra, i.e. mathematical combination of a raster grid cell by cell [8]. A combination is proposed in order to evaluate the difference between two density maps. It builds on the methodological advances of the *Archaedyn* program [9]. This method exploits the relative difference in settlement density through the use of Normalised Differential Ratio (*NDR*), which is defined in the same way as the Normalised Difference Vegetation Index, i.e.:

$$\mathrm{NDR}=\;\frac{(T1-T0)}{(T1+T0)}$$

When set against salt resources this cartographic formula allows us to visualise and compare the settlement dynamic between two chronological sequences and thus to distinguish between the areas that are most attractive (from 0.33 to 1), most stable (from − 0.33 to 0.33) and those that have fallen into decline/disuse (from − 1 to − 0.33).

This approach allows a discussion of the main chronological periods in terms of population density and distribution. Correlated with the geographical breakdown of salt resources, these analyses allow a global understanding of land use processes as well as of settlement dynamics. In doing so, this study complements other analytical studies [1].
