# Supplementary material for: Kernel density estimation and transition maps of Moldavian Neolithic and Eneolithic settlement
Source: Data Brief. 2018 Feb 3;17:452–8. doi: 10.1016/j.dib.2018.01.051 (PMC5988340; doi:10.1016/j.dib.2018.01.051)
Supplement: Supplementary file 1 — Transparency document [file mmc1.docx]

**Subject**: Declaration confirming the absence of any conflict of interest related to the paper prepared by Robin Brigand and Olivier Weller and submitted in Data in Brief (Kernel density estimation and transition maps of Moldavian Neolithic and Eneolithic settlement)
